# Systemically administered liposome-encapsulated Ad-PEDF potentiates the anti-cancer effects in mouse lung metastasis melanoma

**DOI:** 10.1186/1479-5876-11-86

**Published:** 2013-04-03

**Authors:** Hua-shan Shi, Li-ping Yang, Wei Wei, Xiao-qing Su, Xiao-peng Li, Meng Li, Shun-tao Luo, Hai-long Zhang, Lian Lu, Yong-qiu Mao, Bing Kan, Li Yang

**Affiliations:** 1 State Key Laboratory of Biotherapy and Cancer Center, West China Hospital, West China Clinical Medicine School, Sichuan University, Chengdu, Sichuan, PR China; 2 State Key Laboratory of Biotherapy and Department of Head and Neck Oncology, West China Hospital, West China Medical School, Sichuan University, Keyuan Road 4, Chengdu, Sichuan, PR China; 3 Department of Oncology, The First Hospital of Lanzhou University, Lanzhou, Gansu, PR China

**Keywords:** PEDF, Adenovirus, Cationic liposome, Melanoma, Gene therapy

## Abstract

**Background:**

The use of adenoviral vector for gene therapy is still an important strategy for advanced cancers, however, the lack of the requisite coxsackie-adenovirus receptor in cancer cells and host immune response to adenovirus limit the application of adenoviral vector in vivo.

**Method:**

We designed the antiangiogenic gene therapy with recombinant PEDF adenovirus (Ad-PEDF) encapsulated in cationic liposome (Ad-PEDF/Liposome), and investigated the anti-tumor efficacy of Ad-PEDF/Liposome complex on inhibition of tumor metastasis.

**Results:**

We found that systemic administration of Ad-PEDF/liposome was well tolerated and resulted in marked suppression of tumor growth, and was more potent than uncoated Ad-PEDF to induce apoptosis in B16-F10 melanoma cells and inhibit murine pulmonary metastases in vivo. After Ad-luciferase was encapsulated with liposome, its distribution decreased in liver and increased in lung. The anti-Ad IgG level of Ad-PEDF/Liposome was significantly lower than Ad-PEDF used alone.

**Conclusion:**

The present findings provide evidences of systematic administration of cationic liposome-encapsulated Ad-PEDF in pulmonary metastatic melanoma mice model, and show an encouraging therapeutic effect for further exploration and application of more complexes based on liposome-encapsulated adenovirus for more cancers.

## Background

Melanoma is a tumor of transformed melanocytes; and it is a potentially serious type of skin cancer [1], which is one of the most highly invasive and metastatic tumors. Malignant melanoma is an increasingly common malignancy, and its mortality rates have been rapidly increasing above those of any other cancer in recent years [2,3]. Melanoma can spread "silently" at an early stage without any symptoms of metastasis, and owing to the incidence of melanoma is increasing in last decades, the mortality rate of melanoma is still increasing [3]. Thus, it is imminent to seek new strategies for treating patients with melanoma who are at high risk of metastasis.

Angiogenesis plays a critical role in the process of growth and metastasis of primary solid tumors [4,5]. The endothelial cells are genetically stable and have no resistance via repeated administration [6-8]; so anti-tumor therapy is aimed at endothelial cells by inhibiting neovascularization and interrupting blood supplication for tumor, which could slow down the tumor growth [9,10]. The current review summarizes existing knowledge of the mechanisms of angiogenesis in melanoma [11], and current anti-angiogenic therapeutic strategies and their targets confirmed the effect of anti-angiogenic therapy on melanoma [12-15].

Pigment epithelium-derived factor (PEDF) is a 50-kDa protein isolated from conditioned media of the retinal pigment epithelial cells as a potent endogenous inhibitor of angiogenesis [16]. PEDF could inhibit the proliferation and migration of endothelial cells toward many angiogenic inducer, including platelet-derived growth factor, vascular endothelial growth factor (VEGF), interleukin-8, acidic fibroblast growth factor, and lysophosphatidic acid [17], and then suppress angiogenesis. PEDF could prevent melanoma growth via angiogenesis inhibition [2,18]. The lack of PEDF expression may contribute to the pathogenesis of malignant melanoma [19]. Therefore, over expression of PEDF could inhibit angiogenesis and the growth of malignant melanoma cells [18]. However, there are some setbacks in clinical application with PEDF due to difficulties and the high cost of producing large quantities of biologically active proteins and the short half-life of PEDF [17]. Gene therapy offers a preferable pathway to solve these problems.

Adenoviral vector (Ad) is the widely utilized vehicle for gene transfer in a variety of gene therapies, because they can transfect many cell types [20-23]. However, due to the innate immunogenicity of adenovirus and its targeting cellular receptor dependency, such as Coxsackie-adenovirus receptor (CAR), the therapeutic effect of gene transfer therapy decreases. In addition, no better effect could been gained by repeating administration [24,25], as drugs only accumulate in the liver other than transport to other normal tissues when intravenous administration of an adenovirus vector [26,27]. Fortunately, recent studies suggest that Ad encapsulated with liposome may be an effective strategy to escape the neutralization caused by immune response and enhance gene transfer [28,29]. Given these, we studied that gene delivery liposome encapsulating adenovirus-encoding PEDF may be more efficient and safer treating strategy for improving gene therapy.

In this study, we used anti-angiogenesis with gene therapy by developing PEDF encoding adenovirus; and then we used cationic liposome which was composed of (1, 2-dioleoyloxypropyl)-N, N, N-trimethy-lammonium chloride DOTAP: chol (cholesterol) to encapsulate the recombined adenovirus-encoding PEDF. We investigated the antitumor activities of the intravenous administration of cationic liposome-encapsulated recombinant PEDF adenovirus in C57BL/6 mice model that were planted with B16-F10 melanoma cells. Our study indicates that the complexes can not only be safe to recipient mice, but also generate enhanced and lasting antitumor effects in vivo, and makes for further clinical application.

## Methods

### Cell lines and animals

B16-F10 cell line and the human embryonic kidney (HEK293) cell line were obtained from ATCC (American Type Culture Collection, Manassas, VA), and cultured in RPMI-1640 or DMEM medium (Gibco/Invitrogen, Carlsbad, CA) supplemented with 10% fetal bovine serum (FBS) plus ampicillin and streptomycin, and incubated in a 5% CO_2_, 37°C incubator routinely. C57BL/6 mice (6–8 weeks old) were purchased from the Laboratory Animal Center of Sichuan University. All experiments were approved by the Institutional Animal Care and Treatment Committee of Sichuan University (Chengdu, China).

### Recombinant adenoviral vector construction and viral preparation

A recombined adenovirus carrying PEDF gene (Ad-PEDF) was prepared as previously described, Ad-luciferase and Ad-Null was prepared as the construction of Ad-PEDF, except luciferase gene or no objective gene was inserted [30]. After Ad-PEDF, Ad-luciferase and Ad-Null was constructed, the viral particles were amplified in HEK293 cells, which were maintained in DMEM medium with 10% FBS plus ampicillin and streptomycin, and incubated in a 37°C humidified chamber with 5% CO_2_ atmosphere. The harvested viral particles from the cultures were purified by two-step cesium chloride (CsCl) gradient ultracentrifugation and measured by absorption (A260). The virus titer was quantified using a standard TCID50 assay.

### Preparation of cationic liposome

Cationic liposome was prepared as follows. Briefly, cholesterol and DOTAP (Sigma, USA) were mixed at a molar ratio of 1:1. Then trichloromethane and methanol were added into the mixture at volume ratio of 3:1. After rotary evaporation to remove the organic solvents and vacuum dehydration, the dried lipid film was hydrated with distilled water. Then the lipid film was ultrasonicated to form liposome in ice bath and restored in 4°C.

### Test of Ad/liposome complexes with transmission electron microscope (TEM)

The Ad-liposome complexes were visualized under an electron microscope by using a negative stain. Before TEM (H7650, Hitachi, Japan) observation, Ad/Liposome complexes were incubated for 30 min at room temperature, dripped on copper screens covered with nitrocellulose, followed by phosphotungstic acid for negative staining and drying in room temperature.

### Preparation of adenovirus-liposome complex and determine the particle size

Fixed amount of adenovirus was added into liposomes, the suspension was gently mixed and incubated for 30 min at room temperature. The particle diameter was analyzed using a particle size analyzer (Mastersizer 2000, USA) after the Ad-liposome complexes were performed. The resulting Ad-liposome complexes were prepared fresh at room temperature.

### Optimization of liposome and Ad vector ratio and transfection in B16-F10 melanoma cells by the Ad-GFP/liposome complexes

B16-F10 melanoma cells were transfected with different liposome and Ad-GFP ratios. In brief, Cells were seeded into 6-well plates at a density of 5 × 10^5^ cells per well and used at 70–80% confluence. Various amounts of liposomes (2, 5, 10, 15, 20 μg, respectively) were incubated with a fixed amount of Ad-GFP for 30 min at room temperature, and then added to B16-F10 melanoma cells. At 72 h after infection, the percentage of GFP expressing was determined by Flow Cytometry (FCM) (Epics Elite ESP, Beckman Coulter, USA).

### Effect of neutralizing the antibody in vitro for B16-F10 melanoma cells

Neutralizing antibodies of Ad vector was prepared as before [31]. After obtained the neutralizing antibodies, complexes of Ad-GFP and DOTAP and cholesterol cationic liposomes were used to infect B16-F10 melanoma cells with or without the serum containing the neutralizing antibody at 37°C for 4 h. Then, cells were washed with phosphate-buffered saline to remove the complexes. Another 2 ml fresh medium with 10% FBS was added. The expression of GFP in infected cell samples were visualized with an Olympus microscope at × 200 magnification after Cells were incubated for an additional 48 h.

### Expression of human PEDF carried by the Ad-PEDF/liposme complexes in B16-F10 melanoma cells

The procedure was performed as described previously [30]. Briefly, 5 × 10^5^ B16-F10 melanoma cells were plated into 6-well plates and used at 70%–80% confluence. Then the cells were infected with the Ad-PEDF/Liposome complexes (at a ratio of 5 × 10^7^ i.f.u. Ad-PEDF with 10 μg Liposome), Ad-PEDF or Ad-null. Multiplicity of infection (MOI) of each groups were 500. After infection for 72 h, supernatants were collected and stored at -80°C for western blotting analysis. The proteins of supernatant were separated by SDS-polyacrylamide gel electrophoresis (PAGE) and electronically transferred onto a polyvinylidene difluoride membrane (PVDF, Bio-Rad, Richmond, CA, USA). The blots were probed with a mouse anti-human PEDF monoclonal antibody (3:1000, mAb; R&D Systems, Boston, MA, USA) and a peroxidase-conjugated secondary antibody, goat anti-mouse IgG (1:10,000, ZSGB-BIO, Beijing, China). The protein bands were visualized by an enhanced chemiluminescence (ECL) detection system (Pierce, Rockford, IL, USA).

### Detect of apoptosis of B16-F10 melanoma cells in vitro

B16-F10 melanoma cells were plated in 6-well plates with 5 × 10^5^ cells/well and cultured to 70–80% confluence at 37°C in humid air containing 5% CO_2_. Then, cells were infected with Ad-PEDF/Liposome complexes (at a ratio of 5 × 10^7^ IFU. Ad-PEDF with 10 μg Liposome), Ad-PEDF or Ad-null. Multiplicity of infection (MOI) of each groups were 500. After infection for 72 h, the efficiency of apoptosis for B16-F10 melanoma cells was assayed by Flow Cytometry.

### Luciferase assay for Ad-luc/liposome distribution

The viral distribution was analyzed by using the luciferase reporting system. In brief, on day 1, all the mice were randomly assigned into 4 groups and each group contained 3 mice. Experimental group received 1 × 10^8^ IFU. Ad-luc plus 20 μg liposome while the control groups received 1 × 10^8^ IFU. Ad-Null and 1 × 10^8^ IFU. Ad-luc (Ad-luciferase) in 0.1 ml normal saline (NS) via i.v. injection, respectively. Seven days later, the mice were sacrificed. Hearts, livers, spleens, lungs, and kidneys from each mouse were collected and individually stored in liquid nitrogen. Using a luciferase assay system kit (Promega, Madison WI, USA), we analyzed luciferase expression in each type of collected organs, according to the manufacturer’s instructions. Briefly, the same organs from the same group were pooled and ground to a fine powder in a mortar containing liquid nitrogen. The fine powder was dissolved and further processed in CCLB solution in the assay kit. The resultant supernatants were collected and subjected to determination of relative light units (RLU, synergy 2, Biotek, Germany), along with a group of standard samples in the kit. The concentration of luciferase in each sample was calculated on the basis of the standard curve.

### Detection of Ad antibodies concentration in sera

On the other hind, we detected Ad antibodies concentration in the sera to assess whether encapsulation of liposome can reduce Ad clearance by systemic circulation. In brief, Ad-Null was diluted to 1 × 10^9^ IFU /ml by 50 mM carbonate coating buffer (PH9.6). 100 μl diluted Ad-Null was added to every well of 96-well ELISA plate and the plate was put at 4°C for overnight. After washing, 150 μl, 1% BSA was added to the plate and blocked at 37°C for 1 hour. Then, after washing, 100 μl different dilution at multi proportion of sera with positive and negative controls were added and incubated at 37°C for 90 minutes. After washing, 100 μl goat anti-mouse Ab tagged horseradish peroxidase (HRP, 1:5000) was added and incubated at 37°C for 1 hour. After washing, TMB (3,3´,5,5´tetramethylbenzidine) as chromogenic agent was added and incubated for 20 minutes at 37°C, followed by 0.5 M sulphuric acid as stop solution. The absorbance was read immediately at 450 nm in a micro-plate reader (3550-UV,BIO-RAD, USA) and the data was used for statistical analysis. There were the serum samples mixed form 3 mice in each group, and each sample were applied to 3 replicated wells.

### Animal models and in vivo effects of Ad-PEDF/liposome

Six to eight week-old female C57BL/6 mice were permitted one week to acclimate to their environment before manipulation. B16-F10 melanoma cells 5 × 10^5^ were injected into C57BL/6 mice by tail vein in 100 μl phosphate-buffered saline. Five days later, mice were randomly divided into four groups: (1) Ad-PEDF plus liposome group (Ad-PEDF/Lipo), i.v. administration of 1 × 10^8^ IFU recombinant adenovirus plus 20 μg liposome; (2) Ad-PEDF group (Ad-PEDF), i.v. administration of 1 × 10^8^ IFU recombinant adenovirus; (3) Ad-null plus liposome group (Ad-null/Lipo), i.v. administration of 1 × 10^8^ IFU plus 20 μg liposome; and (4) normal saline group. To observe therapeutic effects, all of the treatments were performed once. Mice were sacrificed when become moribund by day 18 in normal saline group and lungs from sacrificed mice were removed, the weight and metastasis nodules of lungs were collected; then lungs were fixed in 4% formaldehyde solution for immunochemistry staining and histological analysis. To observe survival time, mice were inoculated in the same way as above (n = 10 per group) and treated once per week for 6 weeks; residual mice in all groups were killed 90 days after tumor establishment.

To determine the effect of anti-angiogenesis treatment of Ad-PEDF/Liposome, frozen tissues were sectioned (5 mm) and fixed in acetone at 4°C. Sections were probed with a monoclonal anti-CD31 antibody and a secondary goat antibody (tetramethylrhodamine isothiocyanate TRITC, dilution: 1:200). Then, the sections were visualized and microvessels were calculated with an Olympus microscope at × 200 magnification.

Furthermore, the apoptotic cells within the metastatic site of B16-F10 melanoma tissue were evaluated by a commercially available TUNEL kit (Promega, Corporation, Madison, WI, USA), according to the manufacturer’s protocol.

### Statistical analysis

SPSS 13.0 (SPSS Inc., USA) was used for statistical analysis. ANOVA was used to determine statistical significances in the comparisons in this study. The difference is considered as significant if *p* < 0.05.

## Results

### Morphological characteristic of Ad-liposome complexes

**Figure 1:**
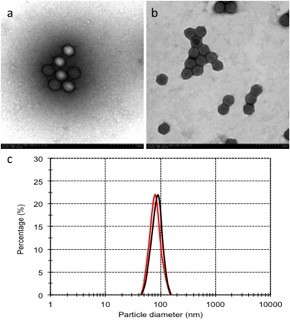
**Particle diameter and TEM photo of DOTAP and cholesterol liposome-encapsulated Ad.** The complex of Ad and liposome was visualized by a transmission electron microscope. The naked adenovirus (**a**) and adenoviral particles encapsulated by DOTAP and cholesterol liposome (**b**). Both the Ad/Liposome complex and Ad alone shows average particle diameter of the particles, red line represent for Ad alone; black line represent for Ad/Liposome complex (**c**).

The complexes of liposome and Ad were verified by a transmission electron microscope. As the photos of TEM showed, there was nothing around the naked Ad as control (Figure 1a), while the adenoviral particles were evenly encapsulated within the liposome (Figure 1b). The size of the complexes was between 80–120 nm and the adenoviral particles were evenly distributed within the DOTAP and cholesterol liposome (Figure 1c).

### Encapsulation of liposome enhances transfection efficiency of Ad in vitro

**Figure 2:**
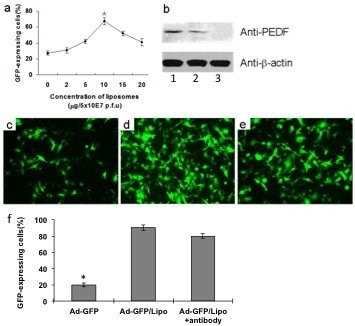
**The Ad/Liposome complex shows enhanced transfection capacity in B16-F10 cells.** B16-F10 cells were transfected at different liposome and Ad-GFP ratio. After infected for 48 h, the percentage of GFP expression was determined by FACS analysis (**a**). Three replicates were performed for each treatment. The data shown are mean ± s.d. (*P < 0.05). Expression of PEDF protein in B16-F10 cells was determined by western blot (**b**). Lane1: transfected with Ad-PEDF liposome complex (Ad-PEDF/Liposome) at a ratio of 5 × 10^7^ IFU/10 μg; lane2: transfected with Ad-PEDF alone (Ad-PEDF); Lane3: untreated. Then, B16-F10 cells were transfected with Ad-GFP (**c**) and Ad-GFP/Liposome (**d**) at a ratio of 5 × 10^7^ IFU/10 μg. Besides, neutralizing antibodies of Ad vector pre-incubated B16-F10 cells were also transfected with Ad-GFP/Liposome complex at the same concentration (**d**). Ad-GFP/Liposome complex transfection noticeably enhanced the efficacy of Ad than that in Ad-GFP transfected B16-F10 cells. However, neutralizing antibodies against Ad vector only partially downregulated GFP expression (**e**) and data are expressed as percentages (**f**). Bars, SD; columns, mean.

The recombined adenovirus-encoding GFP were generated to detect transfection efficiency for B16-F10 melanoma cells. We tried to use fixed amounts of Ad-GFP to incubate with varying concentrations of liposome and then infected B16-F10 melanoma cells, deficiency of CAR [32](data not shown). We found that Ad-GFP encapsulated with DOTAP and cholesterol liposome shows increased transfection ability in B16-F10 melanoma cells. GFP expression increased and then reached a peak with increasing concentrations of DOTAP and cholesterol liposome within 10 μg and then Ad-GFP expression was decreased with increasing concentrations of DOTAP and cholesterol liposome (Figure 2a). On the basis of these data, we selected liposome: Ad-GFP ratios of 10 μg: 5 × 10^7^ IFU for further studies. Then, we detected expression of PEDF in B16-F10 melanoma cells transfected by the Ad-PEDF/Liposome complexes for 72 h using western blotting analysis. As shown in Figure 2b, PEDF was measured in supernatant from B16-F10 melanoma cells infected by Ad-PEDF/Liposome, Ad-PEDF or Ad-null. Obviously, the expression of PEDF transfected by the Ad-PEDF/Liposome complexes was much higher than that of Ad-PEDF and there was noting in Ad-null infected cells. These results indicated that the Ad-PEDF/Liposome complexes could increase the expression of PEDF in B16-F10 melanoma cells.

Then, we used DOTAP and cholesterol liposome-encapsulated Ad-GFP to infect B16-F10 melanoma cells with the existence of Ad-neutralizing antibody. The results showed that the pre-existing neutralizing antibody failed to block the DOTAP and cholesterol liposome-encapsulated Ad-GFP infection to B16-F10 melanoma cells (Figures 2c-e). These findings indicated that the DOTAP and cholesterol liposome-encapsulated Ad-GFP can infect CAR-negative cells in a CAR-independent way.

### Enhancement of apoptosis in B16-F10 melanoma cells by Ad-PEDF/liposome complexes

**Figure 3:**
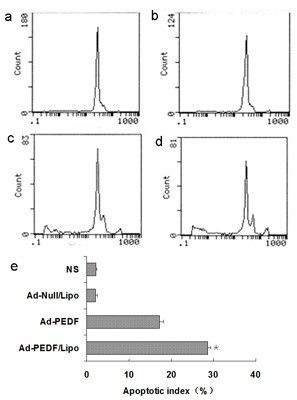
**Apoptosis of B16-F10 cells induced by Ad-PEDF/Liposome in vitro. **Apoptosis cells were determined by flow cytometry. The groups are as follow: (**a**) NS (normal saline), (**b**) Ad-Null/Liposome, (**c**) Ad-PEDF, (**d**) Ad-PEDF/Liposome. Data are expressed as percentages (**e**). The Ad-PEDF/Liposome group showed a significant increase in apoptosis compared with other groups (*P < 0.01). Bars, SD; points, mean (n = 3).

We determined apoptosis in B16-F10 melanoma cells infected by Ad-PEDF/Liposome complexes for 72 h using Flow Cytometry. As shown in Figure 3, there were almost no apoptotic cells in the control and Ad-Null group while a low sub-diploid peak can be seen in Ad-PEDF treated group and the sub-diploid peak became higher with the ratio of Ad-PEDF/Liposome complexes treated group at 10 μg: 5 × 10^7^ IFU. These results indicated that Ad-PEDF encapsulated with liposome could enhance apoptosis, because liposome can improve infection efficiency of Ad-PEDF in B16-F10 melanoma cells, with the result of more expression of PEDF to cause more apoptosis of B16-F10 melanoma cells.

### Encapsulation of liposome changed the distribution of Ad in vivo

**Figure 4:**
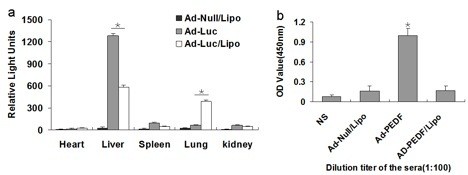
**Detection of host response followed systemic administration of Ad-luc/Liposome.** (**a**) The expression of luciferase of the control groups was high in liver and low in other tissues. However, the luciferase of Ad-luc/Liposome expressed much higher in lung, and significantly lower in liver (*P < 0.01). There was no notable difference in other tissues such as heart, kidney and spleen (*P* > 0.05). (**b**) Specific anti-Ad antibodies in sera. The level of specific anti-Ad immunoglobulins present in the serum of each group was evaluated by ELISA. The values showed that anti-Ad antibodies in Ad-Null/Liposome and Ad-PEDF/Liposome groups are significantly decreased than Ad-PEDF (*P < 0.01). Bars, SD; columns, mean (n = 5).

We determined the distribution of Ad encapsulated with liposome by using the luciferase reporting system. As shown in Figure 4a, we found that the expression of luciferase of the control groups was high in liver and low in other tissues. However, the luciferase of Ad-luc/Liposome expressed much higher in lung, and significantly lower in liver. There was no notable difference in other tissues such as heart, kidney and spleen. This indicated that Ad encapsulated with liposome could change the distribution of Ad in vivo, resulting in decreased clearance of Ad by liver and increased gene expression efficiency to lung.

### Encapsulation with liposome of Ad-PEDF reduces Ad antibody

For detection of Ad antibody response of Ad-PEDF/Liposome complexes, we collected the sera from the treatment groups and controls and measured the Ad antibody concentration in the sera of each group. As Figure 4b shown, on day 20 after B16-F10 melanoma cells injection, at the dilution titer of 1:100, Ad antibody level of Ad-PEDF/Liposome or Ad-Null/Liposome group was significantly lower than that of Ad-PEDF group. This result suggested that the availability of repeated and systemic administration with Ad-PEDF/Liposome could effectively reduce the amount of Ad antibody and inhibit angiogenesis.

### Encapsulation of liposome enhances antitumor effects of Ad-PEDF in vivo

**Figure 5:**
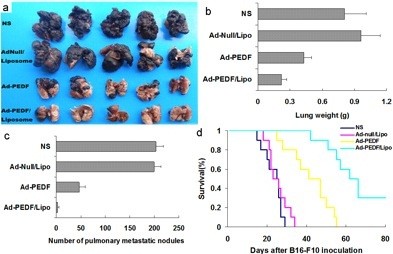
**Anti-cancer effects of Ad-PEDF/Liposome in vivo. **(**a**) Antitumor effects. Tumor-bearing mice were treated with NS, Ad-Null/Liposome, Ad-PEDF and Ad-PEDF/Liposome. There was a significant difference in lung metastasis between PEDF (Ad-PEDF and Ad-PEDF/Liposome) treated group and other groups in both number of pulmonary metastatic nodules (**b**) and lung weight (**c**) (*P* < 0.01). There was also a difference between the Ad-PEDF group and Ad-PEDF/Liposome group (*P* < 0.05). Bars, SD; points, mean (n = 5). A significant increase in survival was found in Ad-PEDF/Liposome-treated mice (**d**) (n = 10, *P* < 0.05, log-rank test).

After confirmation of the success for Ad encapsulated with liposome transfer and expression in vitro, as well as increasing gene expression to lung in vivo, we examined the antitumor efficacy of Ad-PEDF/Liposome treatment in a murine pulmonary metastases model. Figure 5a shows representative images of pulmonary metastases of B16-F10 melanoma in each treatment group. It was obvious that the mice treated with the Ad-PEDF/Liposome complexes bore less pulmonary metastatic nodules than those of mice in other groups. The inhibitory effect on metastases of Ad-PEDF/Liposome complexes was reflected in a statistically significant reduction in weight of the lung compared to other groups. The weight of the lungs from each group is presented in Figure 5b. The mean weight in Ad-PEDF/Liposome group was 0.27 g, 0.43 g in Ad-PEDF group, 0.98 g in Ad-Null/Liposome group and 0.82 g in NS group, respectively. Moreover, the pulmonary metastatic nodules in each mouse were numbered, and results are presented in Figure 5c. From day 20 after B16-F10 melanoma cells inoculation, the number of lung nodules in Ad-PEDF/Liposome treated mice showed significant differences from those in controls (*p* < 0.05). The number of lung nodules in the Ad-PEDF/Liposome treated group was 5 and the metastatic tumors above 3 mm occupied 1.3%, in contrast to 44 in Ad-PEDF group, 200 in Ad-Null/Liposome group, and 200 in normal saline group, in which the metastatic tumors above 3 mm accounted for 12.17%, 20% and 22%, respectively. These results manifest that Ad-PEDF encapsulated with liposome can efficiently inhibit the growth of pulmonary metastases of B16-F10 melanoma in vivo. Survival time with the complex treatment group was significantly longer than other groups including the Ad-PEDF group (Figure 5d).

**Figure 6:**
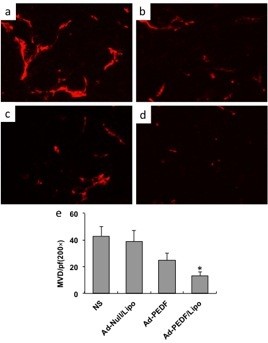
**Inhibition of tumor angiogenesis by Ad-PEDF systemic administration. **Tumors were harvested from NS (**a**), Ad-Null/Liposome (**b**), Ad-PEDF (**c**), or Ad-PEDF/Liposome (**d**) treated mice. Frozen tissue sections were fixed and treated with anti-CD31 antibody. The stained sections were visualized. Significantly decreased micro-vessels were observed in the Ad-E/Lipo-treated group compared with other groups (**e**, *indicates P < 0.05).

To further determine the morphologic changes for the lungs of each treatment group, adjacent sections of lungs were stained with H&E. As shown in Figure 6a, in normal saline group, there were a lot of metastatic tumors with huge volume to oppress the normal tissues of lung and destroy pulmonary alveoli and terminal bronchioles, resulting in pneumorrhagia. In Ad-Null/Liposome group, abundant metastatic nodules occupied normal tissue of lung to induce lung structure reduction and even disappearance. Compared with the controls, in the treatment groups including Ad-PEDF and Ad-PEDF/Liposome complexes, there were sporadic metastatic nodules on the surface of lung, but they were smaller and fewer with normal and clear lung structure in Ad-PEDF/Liposome group. This suggested that encapsulation of liposome can enhance the therapeutic effectiveness of Ad-encoding PEDF on pulmonary metastases of B16-F10 melanoma.

To evaluate the result of anti-angiogenesis therapy, frozen tumor sections were stained with an endothelial-specific antibody against CD31 as before. The decreased micro-vessel densities were found in Ad-PEDF/Lipo-treated tumors. The micro-vessel density was counted in Figure 6.

**Figure 7:**
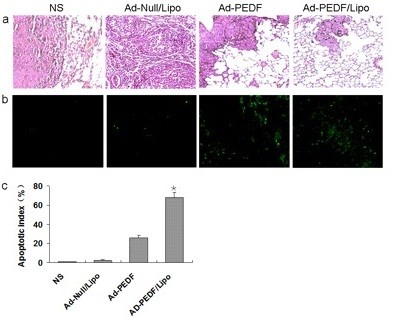
**Histological analysis of tumors. **(**a**) The Hematoxylin and eosin (H&E) staining of lung tissues. After mice were sacrificed histological sections were taken from lungs of mice, and analyzed at × 100 magnification. The mice treated with Ad-PEDF/Liposome showed a significant inhibition of the size of to the metastasis of B16-F10. (**b**) Detection of apoptotic cancer cells in B16-F10 metastasis in C57 BL/6 mice using TUNEL analysis. The percentage of apoptosis was determined by counting the number of apoptotic cells and dividing by the total number of cancer cells in the field (five high power fields/slide). (**c**) Percent apoptosis in each group. The group treated with Ad-PEDF/Liposome resulted in significantly increased apoptosis compared to that of other groups (*P < 0.01). Bars, SD; columns, mean.

To prove whether the anticancer effects of the Ad-PEDF/Liposome complexes associated with the apoptotic cells, TUNEL assay was applied. As shown in Figure 7b-c, there were more green nuclei identified as apoptosis observed in the Ad-PEDF/Liposome complexes treated tumor tissue than those in other treated groups (*p* < 0.05). This suggests that inhibited pulmonary metastases of B16-F10 melanoma by the Ad-PEDF/Liposome complexes may be induced by apoptosis.

### Toxicity observation

**Figure 8:**
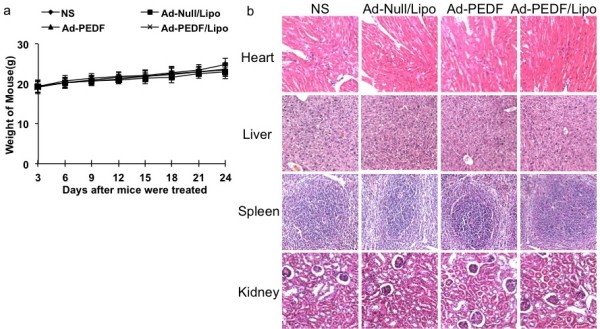
**Toxicity observation. **(**a**) Body weights of mice were plotted and the curve of the Ad-PEDF/Lipo-treated and Ad-null/Lipo-treated group paralleled very closely to that of the Ad-PEDF group and ns group with no significant differences among them (P > 0.05). (**b**) The Hematoxylin and eosin (H&E) staining of heart, liver, spleen and kidney. After mice were sacrificed histological sections were taken from lungs of mice, and analyzed at × 200 magnification. The organs kept primitively normal without inflammatory cells infiltration and necrosis in each group.

To evaluate the health status of mice in each group, the weight of mice was monitored once every 3 days after mice were treated with Ad-PEDF/Lipo (i.v. administration of 1 × 10^8^ IFU recombinant adenovirus plus 20 μg liposome), Ad-PEDF (i.v. administration of 1 × 10^8^ IFU recombinant adenovirus), Ad-null/Lipo (i.v. administration of 1 × 10^8^ IFU plus 20 μg liposome) and normal saline. No significant differences in weights were found among all the groups (Figure 8a). Furthermore, we determined whether encapsulation of liposome had the toxic and side effect on other normal tissues of Ad-PEDF/Liposome complexes with H&E staining. The organs including heart, liver, spleen and kidney kept primitively normal without inflammatory cells infiltration and necrosis (Figure 8b). This result indicated that Ad encapsulated with liposome appeared no obvious toxic reaction to normal tissues.

## Discussion

Melanoma which derived from neuroectoderm has a high malignancy with poor prognosis, due to the vascular and lymphatic metastasis during the late stage [6]. It still remains a major clinical challenge in treating melanoma, and the existing therapeutic protocols are limited. Thus, the development of novel treatment approaches is required [3]. Angiogenesis has been shown to play a key role in tumor growth and metastasis. PEDF plays an important role in the process of angiogenesis of melanoma, which could inhibit endothelial cell proliferation and migration toward many angiogenic inducer [17], and then prevents melanoma growth via angiogenesis inhibition [2,18]. PEDF displays broad anti-tumor activity not only based on targeting of the tumor microenvironment (anti-angiogenic action) but also the tumor cells (direct anti-tumor action) [33]. Loss of PEDF enables migration, invasion and metastatic spread of melanoma [33], and constitutive over expression of PEDF could inhibit melanoma growth and metastasis [34]. So PEDF is an ideal target for treatment of melanoma, and it does have good efficacy on inhibition metastatic spread of melanoma in our study.

PEDF-based gene therapy targets blood vessel-proliferating endothelial cells and cancer cells, however, intravenous and prolonged administration is required for better benefits in clinically administration. In this study, we utilized adenovirus to encode PEDF to inhibit angiogenesis of B16-F10 melanoma, due to its high gene transfection efficiency [35,36]. However the coxsackievirus-adenovirus receptor (CAR) levels are often low in tumor cells and could not be detected in the endothelial cells [37,38]. Therefore, we performed a systemic and repeated administration with cationic liposome-encapsulated adenovirus to explore target and the antitumor effects in pulmonary metastases of B16-F10 melanoma model.

Cationic liposome can not only protect negative macromolecular drugs, but also deliver them to target cells [39-41]. Generally, the binding between adenovirus and targeting cells was dependent on the adenoviral fiber knob with CAR of the cell surface [31]. Cationic liposome can mask adenovirus and increase the efficiency of gene transfer in vitro and broaden vector tropism to various cells and tissues, as well as reduce immunogenicity in vivo [42-44], and thus provides a feasible way to improve the ability of adenovirus to infect the special tissue and cancer cells. Previous investigations have shown that complexation of Ad vectors with cationic lipids can improve infection of cultured cells [45-47]. Moreover, cationic liposome enhanced infection of Ad vectors mainly by improving the cellular uptake, altered bio-distribution and reduced immunogenicity of adenovirus [28,48,49]. In this study, the liposome was composed of DOTAP and cholesterol, which kept liposome’s cationic performance and its stabilization separately. Cholesterol incorporation into DOTAP liposome enhanced stability and uptake of the complexes of Ad vector with liposome and further improved the infection efficiency of CAR-deficient tumor cells [32]. Besides, we prepared the cationic liposome to encapsulate Ad vector and applied to detect bio-distribution of the complexes in vivo. Then we found that significantly more expression in lung and less expression in liver than Ad used alone. This suggested that the cationic liposome could alter the bio-distribution of Ad vector, which was in accord with the previous studies [49-51].

Furthermore, we investigated the anti-cancer effect by gene therapy mediated by the Ad-PEDF/Liposome complexes to pulmonary metastases of B16-F10 melanoma, on the basis of Ad vector encapsulated with cationic liposome increasing gene expression to lung. In vivo, Ad-PEDF/Liposome showed stronger inhibited angiogenesis, metastases of B16-F10 melanoma and increased apoptosis, as well as the growth of metastatic tumors than Ad-PEDF used alone. This result indicated that liposome-encapsulated Ad could prolong the expression of PEDF and there is more secreted transduction and PEDF secretion or because PEDF secretion is localized to the lung/tumor, these are need to be confirmed in future experiments. These findings support that the gene therapy strategy of anti-angiogenesis based on the cationic liposome is feasible for pulmonary metastases of B16-F10 melanoma therapy.

However, there is another problem that adenoviral clearance due to vector immunogenicity in vivo induces the failure of repeated administration of recombinant adenovirus [52]. In our research, Ad antibody levels from complex-injected mice were significantly lower than those from Ad-PEDF injected alone. The cationic liposome has advantage as a physical barrier to Ad vector and protects Ad-PEDF to escape from host immune clearance and extends blood circulation time. Therefore, Ad-PEDF/Liposome complexes can possibly be repeatedly applied via intravenous administration. These results indicated that anti-angiogenesis therapy medicated by Ad-encoding PEDF encapsulated with the cationic liposome profited to reduce tumor angiogenesis and metastases of B16-F10 melanoma. However, the complexes were unable to inhibit tumor growth completely. It is necessary to optimize liposomal formations and therapeutic scheme to gain better therapeutic efficacy.

## Conclusion

In summary, we found advantages in the application of the cationic liposome-encapsulated Ad vectors to gene therapy. Anti-angiogenesis gene therapy mediated by adenovirus with the cationic liposome can be used to synergize with PEDF and further inhibit the growth of B16-F10 melanoma. In the future, further improvements in formation method of the cationic liposome, as well as advancement of Ad vectors will take out to inhibit tumor growth fully and apply to other antitumor models, through optimizing the encapsulation of the cationic liposome with Ad.

## Abbreviations

PEDF: Pigment epithelium-derived factor; VEGF: Vascular endothelial growth factor; Ad: Adenoviral vector; CAR: Coxsackie-adenovirus receptor; Ad-PEDF: Recombined adenovirus carrying PEDF gene; IFU: Infectious unit; TEM: Transmission Electron Microscope; Ad-GFP: Recombined adenovirus carrying GFP gene; FCM: Flow Cytometry; Ad-PEDF/Lipo: Ad-PEDF and Liposome complexes.

## Competing interests

The authors declare that they have no competing interests.

## Authors’ contributions

HS S, LP Y and W W performed experiments, interpreted results, drafted manuscript. XQ S, XP L and M L designed experiments, interpreted results. ST L and HL Z conducted experiment. L L and YQ M interpreted results. B K and L Y made critical revision to manuscript. All authors have read and approved the final manuscript.
